# Air Travel Is Associated with Intracontinental Spread of Dengue Virus Serotypes 1–3 in Brazil

**DOI:** 10.1371/journal.pntd.0002769

**Published:** 2014-04-17

**Authors:** Marcio R. T. Nunes, Gustavo Palacios, Nuno Rodrigues Faria, Edivaldo Costa Sousa, Jamilla A. Pantoja, Sueli G. Rodrigues, Valéria L. Carvalho, Daniele B. A. Medeiros, Nazir Savji, Guy Baele, Marc A. Suchard, Philippe Lemey, Pedro F. C. Vasconcelos, W. Ian Lipkin

**Affiliations:** 1 Centro de Inovação Tecnológica, Instituto Evandro Chagas, Ananindeua, Brazil; 2 Center for Genomic Sciences, United States Army Medical Research Institute for Infectious Diseases, Frederick, Maryland, United States of America; 3 Center for Infection and Immunity, Mailman School of Public Health, Columbia University, New York, New York, United States of America; 4 Department of Zoology, University of Oxford, Oxford, United Kingdom; 5 Departamento de Arbovirologia e Febres Hemorrágicas, Instituto Evandro Chagas, Ananindeua, Brazil; 6 Department of Biomathematics, David Geffen School of Medicine, University of California – Los Angeles, Los Angeles, California, United States of America; 7 Department of Human Genetics, David Geffen School of Medicine, University of California – Los Angeles, Los Angeles, California, United States of America; 8 Department of Biostatistics, UCLA Fielding School of Public Health, University of California - Los Angeles, Los Angeles, California, United States of America; 9 Universidade do Estado do Pará, Belém, Pará, Brazil; Yale University, United States of America

## Abstract

Dengue virus and its four serotypes (DENV-1 to DENV-4) infect 390 million people and are implicated in at least 25,000 deaths annually, with the largest disease burden in tropical and subtropical regions. We investigated the spatial dynamics of DENV-1, DENV-2 and DENV-3 in Brazil by applying a statistical framework to complete genome sequences. For all three serotypes, we estimated that the introduction of new lineages occurred within 7 to 10-year intervals. New lineages were most likely to be imported from the Caribbean region to the North and Northeast regions of Brazil, and then to disperse at a rate of approximately 0.5 km/day. Joint statistical analysis of evolutionary, epidemiological and ecological data indicates that aerial transportation of humans and/or vector mosquitoes, rather than *Aedes aegypti* infestation rates or geographical distances, determine dengue virus spread in Brazil.

## Author Summary

Dengue virus serotypes are associated with millions of infections and thousands of deaths globally each year, primarily in tropical and subtropical regions. We investigated the spatial dynamics of DENV (serotypes 1–3) in Brazil by applying a statistical framework to complete genome sequences. Co-circulation of distinct genotypes, lineage extinction and replacement and multiple viral introduction events were found for all three serotypes. New lineages were typically introduced from the Caribbean into Northern Brazil and dispersed thereafter at a rate of ≈0.5 km/year. Our analysis indicates that aerial transportation is a more important determinant of viral dispersal than *Aedes aegypti* infestation rates or geographical distance.

## Introduction

Dengue fever, caused by the flavivirus *Dengue virus* (DENV), is the most important and widespread arthropod-borne viral disease, causing an estimated 392 million human infections per year [1]. DENV is most prevalent in tropical and subtropical areas of the globe, where ecologic and epidemiologic conditions sustain virus circulation [2]. Myriad factors, including unplanned urbanization, increased numbers of susceptible humans and air travel networks, have likely played an important role in increasing the number of cases and spread of DENV serotypes throughout the developing world [3], [4]. According to the World Health Organization (WHO), there are currently at least three billion people living in more than 100 countries at risk for DENV outbreaks [5].

Several epidemiological and molecular studies suggest that DENV and yellow fever virus were first introduced in the Americas during the early transportation of slaves [6], [7]. The first outbreak in the Americas occurred in Peru in the early 1800s. Outbreaks in the Caribbean islands, United States, Colombia and Venezuela followed thereafter [8]. In Brazil, the first cases were reported in 1845 in the southeastern city of Rio de Janeiro [9]; the first laboratory-confirmed outbreaks were reported in Boa Vista, Roraima State, Northern Brazil in 1982 [10] and later in Rio de Janeiro, 1986 [11]. All four serotypes are currently hyperendemic in Brazil. DENV serotypes 1, 2 and 3 have been circulating in Brazil for at least two decades [12]; DENV serotype 4 has been circulating since at least 2010 [13].

Over the past ten years, several epidemiological and molecular epidemiological studies were conducted to gain a better understanding of DENV evolution and introduction events that contributed to DENV outbreaks in Brazil [14]–[16]. Due to the fast pace of DENV genome evolution, its spatial and evolutionary dynamics occur on the same time scale [4], [17]. Phylogenetic diffusion approaches have been utilized to describe the spatial dynamics of past movement events of DENV evolution, using partial as well as full genome data. We have recently shown that DENV serotype 4 genotype II was introduced from the Northern region of South America and the Caribbean whereas genotype I was introduced from Southeast Asia [13], [15]. However, the origins, establishment and geographical movement of DENV serotypes 1, 2 and 3 remain unclear. Here, we apply a combination of molecular clock, coalescent and discrete phylogeographic models to full genome sequences to deduce the past movements of DENV serotypes 1 through 3 on a global scale, focusing on South American countries. We also use 2D random-walk models that consider viral dispersal in continuous space to estimate the rate of DENV lineage dispersal within Brazil. Finally, we use a recently developed discrete diffusion approach based on generalized linear models to estimate the impact of epidemiological and ecological determinants of DENV dispersal.

## Methods

### Viral strains

A total of 98 DENV strains (34 DENV-1, 33 DENV-2 and 31 DENV-3) isolated from different Brazilian geographic areas were included in this study. Viral isolates corresponded to low-passage virus strains obtained after a single passage history in C6/36 cells. The studied strains were obtained from the World Health Organization/PanAmerican Health Organization Reference Center for Arbovirus Reference and Research at the Department of Arbovirology and Hemorrhagic Fevers, Instituto Evandro Chagas, Brazilian Ministry of Health, Ananindeua, Brazil. Table 1 summarizes the strains used for complete genome sequencing, phylogeographic and spatial-temporal analyses according to serotype, source, state and year of isolation.

**Table 1:** DENV strains used for complete genome sequencing, phylogeographic and spatial-temporal analyses according to its serotype, strain, source of isolation and geographic location (Federal states or cities) in Brazil.

Serotype	Strain	Source of isolation	State of isolation	Year of Isolation	Serotype	Strain	Source of isolation	State of isolation	Year of isolation
	H527543	human	Ceará	1994		H 652413	human	Espírito Santo	2002
	H547625	human	Pará	1996		H 654413	human	Mato Grosso do Sul	2002
	H550175	human	Minas Gerais	1997		H 645487	human	Maranhão	2001
	H551022	human	Pará	1997		H 642152	human	Ceará	2001
	H611377	human	Maranhão	1999		H 626903	human	Roraima	2000
	H622822	human	Mato Grosso	2000		H 629766	human	Acre	2000
	H631185	human	Ceará	2000		H 617724	human	Rio Grande do Norte	1999
	H631188	human	Ceará	2000	**DENV-2**	H 618438	human	Pará	1999
	H628435	human	Acre	2000		H 623360	human	Pará	2001
	H648234	human	Amapá	2001		H628243	human	Pará	2000
	H693852	human	Rio Grande do Norte	2001		H 527541	human	Ceará	1994
	H650290	human	Roraima	2001		H 527821	human	Ceará	1994
	H655243	human	Piauí	2002		H 517822	human	Ceará	1994
	H660409	human	Amazonas	2002		H 533198	human	Minas Gerais	1995
**DENV-1**	H660415	human	Amazonas	2002		H 547176	human	Roraima	1996
	H655251	human	Piauí	2002		H 547177	human	Roraima	1996
	H650975	human	Mato Grosso	2002		H 508744	human	Tocantins	1991
	H656274	human	Tocantins	2002		H 506347	human	Rio Grande do Norte	1991
	H672029	human	Maranhão	2003		H 666426	human	Goiás	2003
	H685572	human	Pará	2004		H 650477	human	Mato Grosso	2002
	H695190	human	Amapá	2005		H 665993	human	Rio Grande do Norte	2003
	H716995	human	Pará	2006		H 666425	human	Goiás	2003
	H739688	human	Amazonas	2007		H 660007	human	Roraima	2002
	H733587	human	Roraima	2007		H 662476	human	Maranhão	2002
	H721251	human	Pará	2007		H 659202	human	Belém*	2002
	AR 721365	*Aedes aegypti*	Pará	2007		H 685606	human	Roraima	2004
	AR 721368	*Aedes aegypti*	Pará	2007		H 675971	human	Amazonas	2004
	H748499	human	Roraima	2008		H 675948	human	Belem	2004
	H 716995	human	Pará	?		H 687202	human	Belem	2005
	H 650290	human	Roraima	?		H 696789	human	Acre	2005
	H 693857	human	Rio Grande do Norte	?	**DENV-3**	H 696735	human	Roraima	2005
	H 672029	human	Maranhão	?		H 692798	human	Roraima	2005
	H741571	human	Mato Grosso do Sul	2008		H 692808	human	Roraima	2005
	H745526	human	Espirito Santo	2008		H 692262	human	Belem*	2005
	H 745039	human	Rio Grande do Norte	2008		H 702980	human	Amazonas	2006
	H 739202	human	Tocantins	2008		H 704582	human	Maranhão	2006
	H 726377	human	Amapá	2007		H 707629	human	Mato Grosso	2006
	H 723494	human	Maranhão	2007		H 712120	human	Rio Grande do Norte	2006
	H 723495	human	Maranhão	2007		H 707877	human	Mato Grosso	2006
	H 730923	human	Amazonas	2007		H 706777	human	Belem*	2006
**DENV-2**	H 710008	human	Amapá	2006		H 705063	human	Tocantins	2006
	H 710686	human	Rondônia	2006		H 721198	human	Belem*	2007
	H 709119	human	Tocantins	2006		H 734020	human	Rio Grande do Norte	2007
	H 688004	human	Pará	2005		H 734230	human	Roraima	2007
	H 674704	human	Pará	2004		H 724440	human	Tocantins	2007
	H 676618	human	Acre	2004		H 735102	human	Acre	2007
	H 666995	human	Pará	2003		H 724441	human	Tocantins	2007
	H 660059	human	Amapá	2002		H 741675	human	Roraima	2008
	H 655259	human	Piauí	2002		H 740416	human	Amazonas	2008

*City of Belém, capital of Pará State, Northern Brazil;

?: year of isolation not provided.

### Whole genome sequencing

The complete genome sequences for each DENV serotype were obtained as previously described [15]. To avoid redundancy in methodology, only essential aspects are described. The entire Open Reading Frames were completed using the GS 454 platform [18] and the 5′ and 3′ untranslated regions (UTR) were amplified using a specific set of primers (see Table S1), cloned into the TOPO TA cloning plasmidial-bacterial system (Invitrogen, Carlsbad, CA, USA), and then sequenced in both directions using the plasmid M13F/M13R primers, the ABI Prism BigDye Terminator v3.1 Sequencing Kit (Life Technologies, Foster City, CA, USA), and the ABI 3500 XL sequencer (Life Technologies, Foster City, 92 CA, USA).

### Genome assembly

The DENV (DENV-1 to DENV-3) genomes were obtained by assembling reads generated by both GS FLX 454 System and ABI 3500 XL sequencers. The Mapping reference method implemented in the gs-mapper program, available in Newbler v.2.6 software (Data Processing Software Manual 454 Life Science, 96 http://www.454.com/) was conducted using the following parameters: input, 20 bp; all contig threshold, 100; large contig threshold, 200; minimum overlap length, 40; minimum overlap identity, 70%; k-mer, 12 (seed step), and k-mer, 16 (seed length). The mapping reference strategy was used to reorganize the reads against previous selected reference sequences (for DENV-1: FJ850077; DENV-2: FJ850074 DENV-3: KC425219) as representative of the largest genomes available at the NCBI database (http://www.ncbi.nlm.nih.gov). The entire genomes for each DENV isolate (n = 98) were deposited in the GenBank under the accession numbers (provided after acceptation).

### Data selection

The data set used for these analyses consisted on a total of 2,566 complete DENV genomes available at the GenBank database [19] and the 98 new Brazilian DENV complete genomes. Full-length sequences were grouped by serotype and aligned separately using MAFFT software [20]. Manual editing was performed to improve the resulting alignment [21]. The total number of full-length genomes analyzed were 1,232 for DENV-1, 793 for DENV-2 and 639 for DENV-3. For each dataset, a Neighbor-joining tree was constructed using SeaView [22]. To improve computation time for subsequent analyses, a subset of the global diversity of DENV was selected based on genetic diversity and maximization of the sampling interval. This resulted in datasets of 287 DENV-1, 294 DENV-2, and 352 DENV-3 genomes sampled from 1964 to 2010 from a total of 31 distinct countries in Southeast Asia, North America, Central America, the Caribbean and South American countries. No significant evidence of recombination was found using the Phi-test [23] implemented in the SplitsTree4 program [24]. Details of the sequences used in each analysis, along with respective information on the year of isolation, geographic location, and corresponding accession numbers are available in Tables S2 and S3). The map in Figure S1 depicts the geographic locations for the sequences used in this study.

### Evolutionary analysis of Brazilian DENV circulating strains

Evolutionary analyses of dengue virus evolution were performed in BEAST v1.7, a flexible Bayesian framework that incorporates molecular clock models, coalescent models and spatial diffusion models [25]. A relaxed molecular clock with a lognormal distribution [26] was used to model rate variation among the branches of an unknown phylogenetic tree, and a GTR+G substitution model was used to account for among-site rate variation. BEAST runs for the DENV-1, DENV-2 and DENV-3 datasets indicated that the GTR+G strongly outperformed the GTR and the simpler HKY model with a log Bayes factor between 84 and 410 for all model comparisons.

To model changes in the effective population size over time for DENV serotypes circulating in Brazil from 2002–2010, we used a recently described coalescent-based model that has been shown to outperform previous non-parametric coalescent approaches [27]. Specifically, for each serotype, we shared the demographic coalescent-based model among lineages circulating in Brazil while allowing for independent substitution model parameters, clock models and distinct phylogenies for each serotype-specific within country circulating lineage. We compared effective population changes (*Ne*) over time (for all circulating Brazilian lineages belonging to a particular serotype) to information on the number of states where each serotype was present or absent throughout 2002 until 2012 based on data available from the Ministry of Health of Brazil [28], [29].

For evolutionary analyses of serotype-specific intracontinental datasets, Markov chain Monte Carlo (MCMC) chains were run for 150 million states. For analyses of intra-country viral diffusion, serotype specific datasets were run for 50 million steps. In both scenarios, evolutionary parameters and trees were sampled every 10,000 states. To increase computational speed, the BEAGLE library [30], [31] was run together with BEAST [25]. Convergence of the MCMC chains was inspected with Tracer (http://tree.bio.ed.ac.uk). After removing 10% burn-in, maximum lineage credibility (MLC) trees were summarized using TreeAnnotator and visualized using FigTree (http://tree.bio.ed.ac.uk).

### Spatial origins of DENV serotype introductions

Evolutionary and spatiotemporal aspects were assessed for each DENV serotype using discrete and continuous phylogenetic diffusion models [32], [33]. Given time-stamped, geo-referenced nucleotide sequence data, a discrete phylogeographic model will estimate the most probable location for each internal node up until the root of an unknown phylogeny [34]. We have previously reported the use of discrete phylogeographic methods with DENV-4 sequences [13]. A statistical framework of phylogenetic spatial diffusion was implemented in BEAST [25], [33] to determine the temporal phylogeographic patterns of DENV-1, DENV-2, and DENV-3. To explore spatial dynamics on an intracontinental scale, we considered geographical regions outside of South America and within each South American country as discrete locations in the asymmetric phylogeographic model [35], [36] for all DENV serotypes. On a global scale, sequences were assigned to the geographical traits: i) North America (USA and Mexico); ii) Central America (El Salvador and Nicaragua); iii) Caribbean region (British Virgin Islands, Dominican Republic, Jamaica, Puerto Rico, U.S. Virgin Islands, Anguilla Caribbean, Saint Lucia and Trinidad and Tobago); iv) South Asia (Sri Lanka, Bangladesh), and v) Southeast Asia (Brunei, Singapore, Thailand, Malaysia, Viet Nam, Cambodia). To achieve higher spatial resolution within South America, we assigned sequences to Brazil, Colombia, Peru, Venezuela and French Guiana, Paraguay, Argentina as discrete geographical traits. Note that only DENV-1 full genome data was available for French Guiana, Paraguay and Argentina. (Table 1 and Table S3).

**Figure 1:**
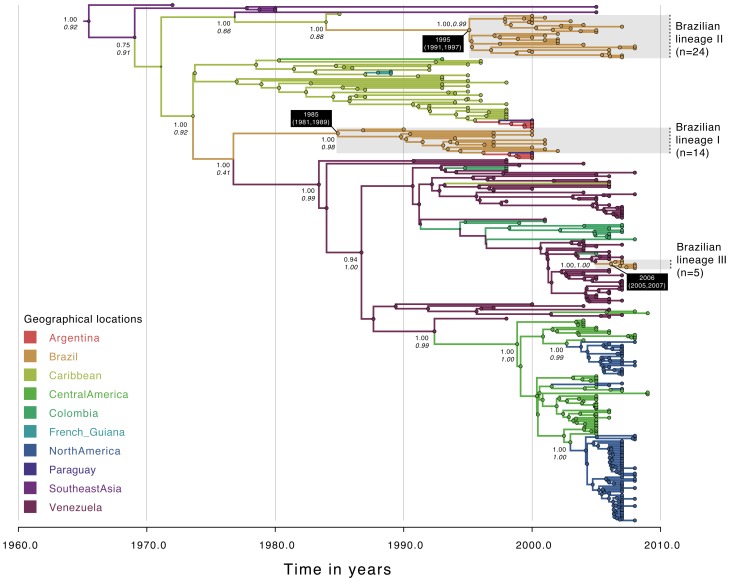
Temporal-scaled phylogeographic DENV-1 tree. Each branch is colored according to the most probable location as inferred using a discrete phylogeographic diffusion model. Geographic locations considered are shown in the left. Phylogenetic posterior probabilities percentages are shown next to relevant nodes along with the location-state posterior support. The number of sequences falling in Brazilian monophyletic lineages (highlighted in grey) is shown in brackets. For each lineage, the mean estimated time of the most recent common ancestor (tMRCA) and respective 95% Bayesian credible intervals (BCI) are shown in a black box.

**Figure 2:**
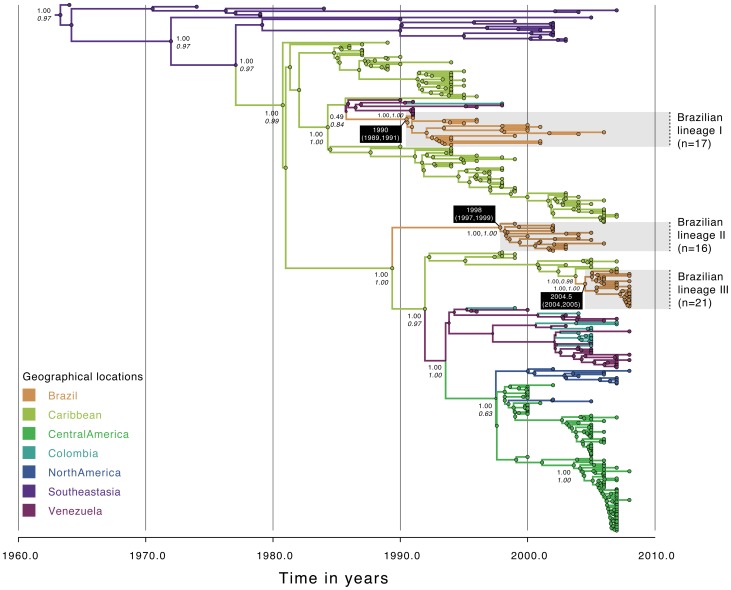
Temporal-scaled phylogeographic DENV-2 tree. Each branch is colored according to the most probable location as inferred using a discrete phylogeographic diffusion model. Geographic locations considered are shown in the left. Phylogenetic posterior probabilities percentages are shown next to relevant nodes along with the location-state posterior support. The number of sequences falling in Brazilian monophyletic lineages (highlighted in grey) is shown in brackets. For each lineage, the mean estimated time of the most recent common ancestor (tMRCA) and respective 95% Bayesian credible intervals (BCI) are shown in a black box.

**Figure 3:**
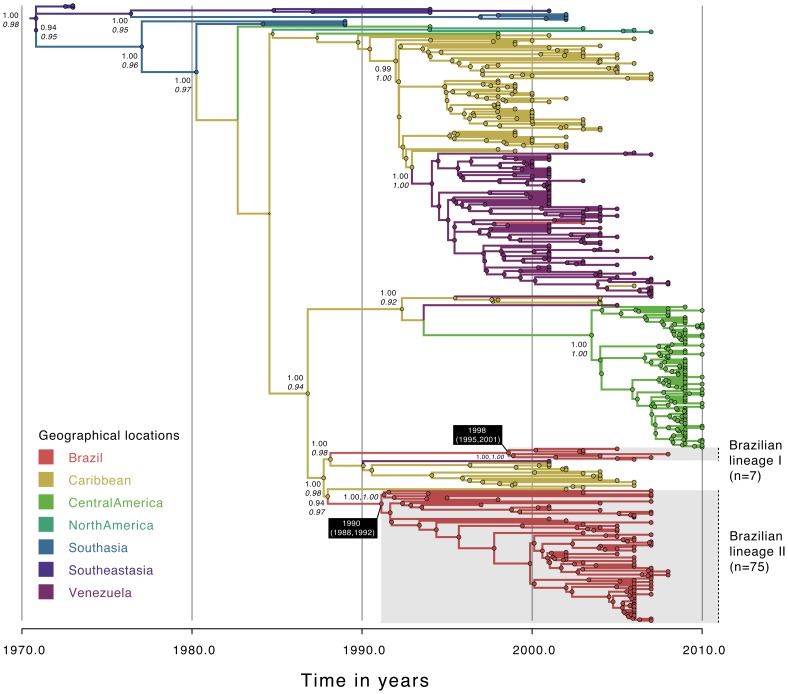
Temporal-scaled phylogeographic DENV-3 tree. Each branch is colored according to the most probable location as inferred using a discrete phylogeographic diffusion model. Geographic locations considered are shown in the left. Phylogenetic posterior probabilities percentages are shown next to relevant nodes along with the location-state posterior support. The number of sequences falling in Brazilian monophyletic lineages (highlighted in grey) is shown in brackets. For each lineage, the mean estimated time of the most recent common ancestor (tMRCA) and respective 95% Bayesian credible intervals (BCI) are shown in a black box.

After identification of Brazilian monophyletic lineages (Figures 1–3), we performed a similar analysis considering only the country-specific lineages circulating in Brazil. In this case, sequence data was assigned to five geographic regions: North, Northeast, Central-West, Southeast and South (see map in Figure S1). Country-specific lineages shared the same instantaneous location-exchange rate matrix. To estimate the most significant pathways of viral dispersal within Brazilian regions, a stochastic search variable selection (BSSVS) procedure was used [32]. A Bayes Factor test was used to identify well-supported migration pathways (log BF >3). The viral dispersal rates identified as significant by the BSSVS procedure were further analyzed by a robust counting procedure [37], [38]. This was used to quantify the number of transitions along the branches of the posterior distribution for source-sink regions involved in well-supported migration pathways.

### Diffusion of DENV serotypes within Brazil

To model spatial diffusion in continuous space and deduce unobserved locations in the entire evolutionary history of within-country circulating lineages (highlighted in Figures 1–3), we only included strains from well-supported lineages circulating in Brazil for which the latitude and longitude were known. Models of continuous diffusion are based on Brownian diffusion models and assume a constant variance random walk along each branch of the phylogeny (homogenous model; no dispersal rate variation). To account for variability along each branch, we used relaxed random walk models (RRW) in BEAST [25], running MCMC chains for 50 million steps and sampling parameters every 50,000^th^ step. Dispersal rates were allowed to vary according to Cauchy, Gamma and Lognormal prior distributions [33]. The performance of the different continuous diffusion models was assessed using stringent model selection procedures available in BEAST. Model selection amongst the different continuous diffusion models was performed using the harmonic mean estimator (HME), as well as its stabilized/smoothed version (sHME), Akaike's information criterion through Markov chain Monte Carlo (AICM) (HME/sHME/AICM equal to 50 million MCMC steps (excluding 20% burn-in), path sampling (PS) and stepping-stone (SS) (64 path steps and a chain length of 1 million steps) [39], [40]. Dispersal rates were reported in units of kilometers per day, along with respective 95% Bayesian Credible Intervals (BCIs). All evolutionary parameters are reported as posterior means along with their 95% Bayesian credibility intervals (BCI). Divergence times and spatial estimates annotated in each MCC tree were converted to a keyhole markup language (KML) file (data provided upon author's request) using the spatial phylogenetic reconstruction of evolutionary dynamics (SPREAD) application [36].

### Identifying potential determinants of DENV diffusion

To investigate the factors driving DENV diffusion we used a recently described generalized linear model (GLM) that parameterizes the logarithm of the instantaneous rate matrix as the logarithm of a combination of a set of epidemiological and ecological predictors [41]–[43]. To evaluate the support and weight of a particular predictor for the dispersal process, we included an inclusion probability and conditional effective size (cES), respectively. Similar to the BSSVS approach [32], the support for each predictor was obtained by comparing the prior with the posterior expectations or the inclusion probability expressed as a Bayes factor.

We tested and quantitated the contribution of epidemiological and ecological predictors to explain countrywide viral dispersal patterns at a regional scale, while reconstructing the evolutionary history and taking into account uncertainty both at the phylogenetic and diffusion level. Toward this aim, we considered: (a) the great circle distances in km that correspond to the shortest geographic distance in km between the centroids representing region; (b) the number of scheduled flights in January 2010 between each pair of regions as obtained from the National Agency of Civil Aviation website (http://www.anac.gov.br); (c) the population density per region (population/square km) as obtained from the Brazilian Institute of Geography and Statistic (www.ibge.gov.br); and (d). the average infestation index of *Aedes aegypti* per geographic region (using data available from the Ministry of Health of Brazil) as a proxy for the number of competent vectors in each region considered. We considered the area of each geographic location in units of km^2^ as an independent predictor (ftp://geoftp.ibge.gov.br/organizacao_territorial/divisao_territorial). To exclude the potential impact of sample sizes in the inference of the factors affecting viral dispersal amongst locations, we also considered the number of sequences in each region. All alignments, BEAST XML and KML files are available upon request.

## Results

### Evolutionary history of DENV genotypes in South America

We determined the consensus genome sequence of 98 Brazilian DENV strains with a mean quality of sequenced bases over 20, mean coverage of 50×, and mean genome length of 10,550 nucleotides. The isolates were from the four geographic regions in Brazil: North (n = 59), Northeast (n = 26), Central-West (n = 9), and Southeast (n = 4) (Table 1).

Evolutionary analysis of 287 DENV-1 full genome sequences showed that Brazilian sequences (n = 42) fell into three distinct lineages, all within genotype V, with a maximum posterior probability (PP) support of 1.00 (Figure 1, Table 2). Numbering of the Brazilian monophyletic lineages is shown in Figures 1–3. The inferred dates for DENV-1 introductions were separated by nearly 10 years, with the first in 1985 (95% BCI: 1981, 1989), the second in 1995 (95% BCI: 1991, 1997) and the most recent in 2006 (95% BCI: 2005, 2007). Whereas both lineage I (n = 24) and lineage II (n = 14) viruses most likely originated in the Caribbean with strong ancestral location PP support (between 0.88 and 0.92), lineage III (n = 5) probably originated in Venezuela (ancestral location PP = 1.00) (Table 2). Sequences from Uruguay (n = 2) and Argentina (n = 7) fell within the same genotype. Our data show two distinct introductions of DENV-1 in Argentina and Paraguay occurring nearly simultaneously (Figure 1), one from the Caribbean (n = 5, ancestral location PP = 0.76) and another from Brazil (n = 4, ancestral location PP = 0.96).

**Table 2:** Mean ages of the MRCA of Brazilian circulating DENV lineages, most probable origins and substitution rates.

Serotype	MRCA (BCI)	Root state location (PP)	Rates (10^−4^ s/s/y) (BCI)
**DENV-1**			
Brazilian lineage I	1985 (1981, 1989)	Caribbean (0.92)	6.97 (5.84, 8.12)
Brazilian lineage II	1995 (1991, 1997)	Caribbean (0.88)	3.26 (2.57, 4.07)
Brazilian lineage III	2006 (2005, 2007)	Venezuela (1.00)	6.41 (2.72, 12.15)
**DENV-2**			
Brazilian lineage I	1990 (1989, 1991)	Caribbean (0.84)	10.03 (8.09, 12.02)
Brazilian lineage II	1998 (1997, 1999)	Caribbean (0.98)	13.99 (11.07, 17.04)
Brazilian lineage III	2004.5 (2004, 2005)	Caribbean (1.00)	14.11 (8.99, 2.04)
**DENV-3**			
Brazilian lineage I	1990 (1988, 1992)	Caribbean (0.97)	3.01 (2.56, 3.48)
Brazilian lineage II	1998 (1995, 2001)	Caribbean (0.98)	2.62 (2.11, 3.23)

Notes: BCI: Bayesian credible interval; PP: Posterior Probability; s/s/y: substitution per site per year. Note that numbering of lineages is convenient and has been ordered according to the estimated time of arrival to Brazil. Dispersal rates estimated according to the best-fit continuous diffusion model (DENV-1: RRW gamma, DENV-2: RRW Cauchy, DENV-3: RRW: Gamma).

The analysis of 294 full genomes of DENV-2 provides clear evidence that at least three distinct viral lineages (maximum PP = 1.00) are circulating in Brazil (Figure 2, Table 2). We infer that lineage I (n = 17) was introduced in 1990 (95%BCI: 1989, 1991), lineage II (n = 16) in 1998 (95%BCI: 1997, 1999), and lineage III (n = 21) in 2004.5 (95% BCI: 2004, 2005). Lineage I was probably introduced from Venezuela or the Caribbean (ancestral location PP = 0.84); lineages 2 and 3 were likely introduced from the Caribbean (ancestral location PP for both lineages is 1.00). Analysis of 352 DENV-3 full genomes revealed the presence of two Brazilian viral lineages (PP = 1.00 for both lineages) (Figure 3, Table 2). Whereas the predominant lineage II (n = 75) emerged in 1990 (95% BCI: 1988, 1992), lineage I (n = 7) emerged around 1998 (95% BCI: 1995, 2001). Both lineages seem to have originated from the Caribbean with strong ancestral location PP support of 0.98 and 0.97 respectively for lineage 1 and 2.

### Population dynamics of DENV lineages in Brazil

**Figure 4:**
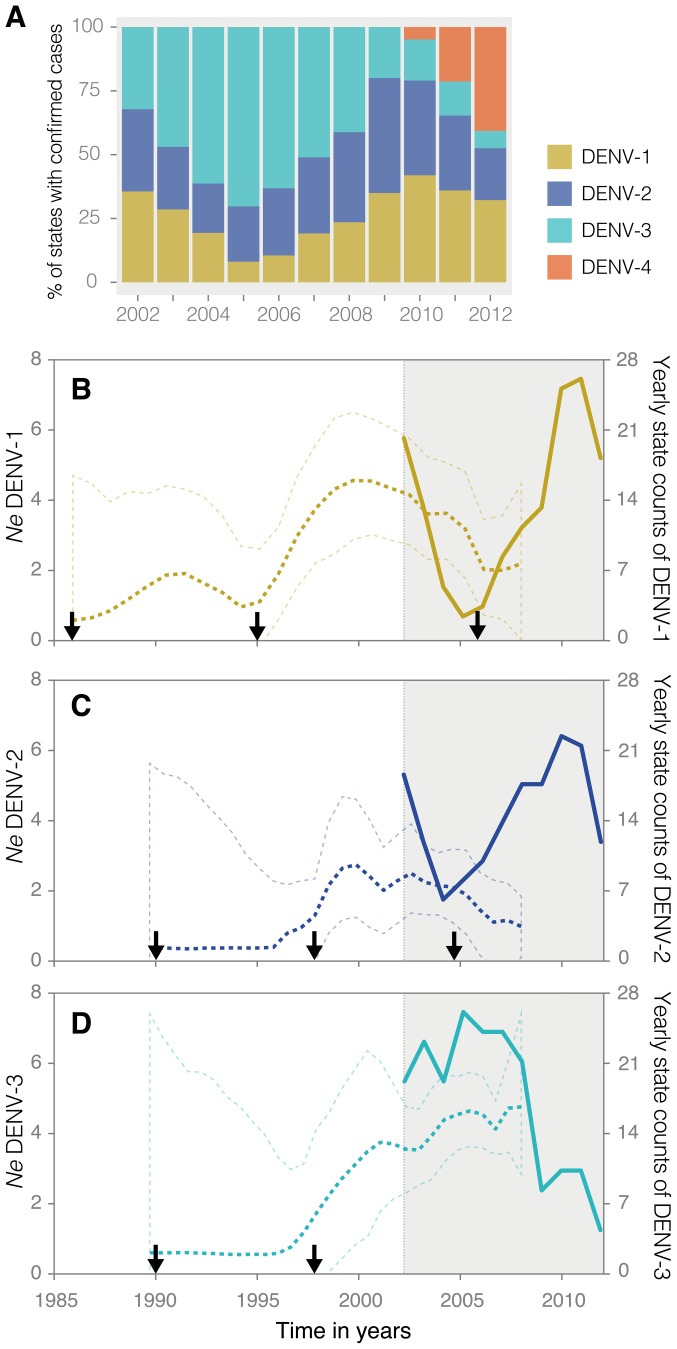
Population dynamics of DENV-1, DENV-2 and DENV-3 circulating lineages in Brazil. Panel A shows the proportion of federal states (total of 27) where each DENV serotype was molecularly confirmed from 2002 to 2012. Panels B, C and D depict changes in effective population size (*Ne*) over time (dashed lines) respectively for DENV-1, DENV-2 and DENV-3 viral lineages circulating in Brazil. Mean estimates of *Ne* (tick dashed line) are shown along with respective uncertainty intervals (thin dashed lines). In panels B–D, filled line shows the yearly counts of federal states where each serotype was detected. The temporal period highlighted in grey corresponds to the time-span for which epidemiological information on serotype-specific state counts was available (2002 to 2012). Data on yearly state counts was available from the Ministry of Health of Brazil [28], [29].

**Figure 5:**
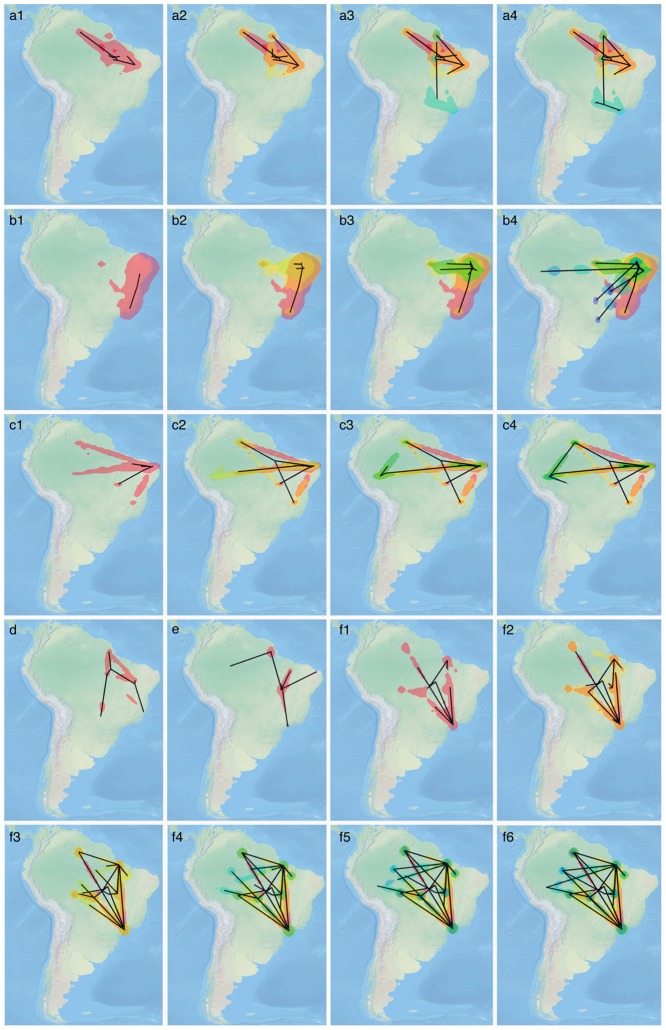
Snapshots of Dengue virus (DENV) lineages spatiotemporal spread. Geographic dispersion of DENV-1 lineage I in 1999 (a1), 2001(a2), 2005 (a3), and 2008 (a4). DENV-1 lineage II: years of 1989 (b1), 1993 (b2), 1995 (b3), and 1997–2001 (b4). DENV-2 lineage I: years of 1992 (c1), 1994–1996 (c2), 1998 (c3), and 2000–2005 (c4). DENV-2 lineage II (d); DENV-2 lineage III (e); DENV-3 lineage II: years of 1998 (f1), 2000 (f2), 2002 (f3), 2004 (f4), 2006 (f5) and 2008 (f6).

We implemented a demographic model to infer the history of dengue virus serotypes in Brazil (see Methods). The proportion of Brazilian federal states (n = 27) in which dengue serotypes were laboratory-confirmed is plotted in Figure 4A. Whereas serotypes 1 and 2 seem to be in-phase, neither is associated with serotype 3. Our demographic reconstruction of serotype dynamics shows a striking concordance between episodes of serotype frequency and the introduction of a new lineage, with the process occurring approximately on a nine-year time scale (estimated time of most recent common ancestors for the different lineages are indicated by arrows, see also Table 2). In serotype 1, ten and eleven years separate the introduction of lineages I, II and III, respectively. The decrease in the number of states where this serotype was detected was correlated with a decrease in detection of lineage II viruses; the later rise in state counts was associated with the introduction of lineage III in 2006 (Figures 1 and 4B). In general, the introduction of a new lineage is associated with a rise in *Ne*. In serotype 2, the estimated time between the introductions of different lineages is eight and approximately seven years, respectively. In this case, it is clear that lineage II was replaced by lineage III (Figure 2), and that the estimated date of introduction of the latter in mid 2004 is strongly associated with a sudden rise in the frequency of states where serotype 2 was reported. In serotype 3, eight years separate the two introductions in Brazil (Figure 4). In this case, both lineages seem to be co-circulating simultaneously (Figure 3, see also Figure 5), although lineage II spread predominantly in South and Northeast Brazil, data on lineage I (mostly from North area), are insufficient to draw significant conclusions. Overall, the results obtained by analyses of genetic data and inferred demographic patterns strongly concur with epidemiological data, indicating that periodic dengue serotype-specific peaks in incidence coincide with the introduction of new lineages in Brazil every 7 to 10 years.

### Spatial dynamics of DENV in Brazil

To investigate the detailed geographic origin of each DENV lineage circulating at a countrywide scale, we included only Brazilian sequences that fell in monophyletic lineages (Figures 1–3) (n = 27 DENV-1, n = 36 DENV-2 and n = 67 DENV-3). Our results obtained by a discrete phylogeographic model indicated that within Brazil, the North region acted as the main hub for viral transmission of DENV to other geographical regions, an observation supported by a statistically significant Bayes Factor of 16.93 (Table 3). More specifically, using a robust counting procedure we found evidence for a total of 10 viral exportations from the North to Northeast region (4 for DENV-1, 3 for DENV-2 and 3 for DENV-3) (Table 3). Three migrations were found from the Northeast to Central-West region (two for DENV-1 and one for DENV-3).

**Table 3:** Most significant links of viral dispersal and mean number of jumps among regions.

Geographic origins		Support	Number of migrations		
From	To	Bayes factor	DENV-1	DENV-2	DENV-3
North	Northeast	16,93	4	3	3
Central-West	Southeast	9,41	-	-	2
Southeast	Central-West	5,08	-	-	1
Northeast	North	3,76	-	-	1
Northeast	Central-West	3,46	2	-	1
South	North	3,3	-	-	1
Southeast	North	3,11	-	-	1

Bayes factor above 3 were considered significant.

### Fast epidemic spread of DENV lineages at a regional scale

Model selection results using the harmonic mean estimator (HME), its stabilized/smoothed version (sHME), Akaike Information Criterion using MCMC (AICM), path sampling (PS) and stepping-stone sampling (SS) for the different distributions underlying continuous diffusion spread for each DENV circulating lineages can be found in Table S4 Overall, the results indicate that relaxed diffusion models provide a better fit than a homogeneous diffusion model, with all model selection approaches preferring the same (gamma-distributed) continuous diffusion model for DENV-1 and DENV-3. The posterior-based estimators (HME, sHME and AICM) prefer this model for DENV-2 as well but are contradicted by the PS and SS estimators, that prefer a Cauchy-distributed continuous diffusion model. Further increases on the computational demands did not yield differing estimates, a sign of convergence of the reported values. To estimate the spatiotemporal dynamics and the spatial rate of diffusion for each serotype-specific DENV lineages, we used the best-fit distributions underlying the continuous diffusion models for each serotype as determined by PS and SS, models that have been shown to outperform HME, sHME and AICM [39], [40]. Table 4 shows the dispersal rate for each circulating lineage while Figure 5 shows the spatiotemporal diffusion of each DENV serotype-specific lineages in Brazil. Our results indicate fast rates of dispersal for each lineage and large heterogeneity in dispersal rates for different lineages, ranging from a minimum of 0.27 km/day (DENV-3, lineage I) to a maximum of 1.17 km/day (DENV-2, lineage III). On average, our results suggest that DENV-2 diffused 1.3 times faster than DENV-1 and 2.5 times faster than DENV-3 (Table 4).

**Table 4:** Dispersal rates of DENV-1, DENV-2 and DENV-3 Brazilian lineages.

Serotype	Dispersal rate (km/d) (BCI)
**DENV-1**	
Brazilian lineage I	0.369 (0.235, 0.520)
Brazilian lineage II	0.663 (0.531, 0.809)
**DENV-2**	
Brazilian lineage I	0.518 (0.345, 0.680)
Brazilian lineage II	0.391 (0.235, 0.607)
Brazilian lineage III	1.178 (0.708, 1.684)
**DENV-3**	
Brazilian lineage II	0.274 (0.207, 0.343)

Dispersal rates in units of km per day (km/d) were estimated according to the best-fitting continuous diffusion model (Supplementary Table S4).

### Human-mediated countrywide dispersal of dengue virus

**Figure 6:**
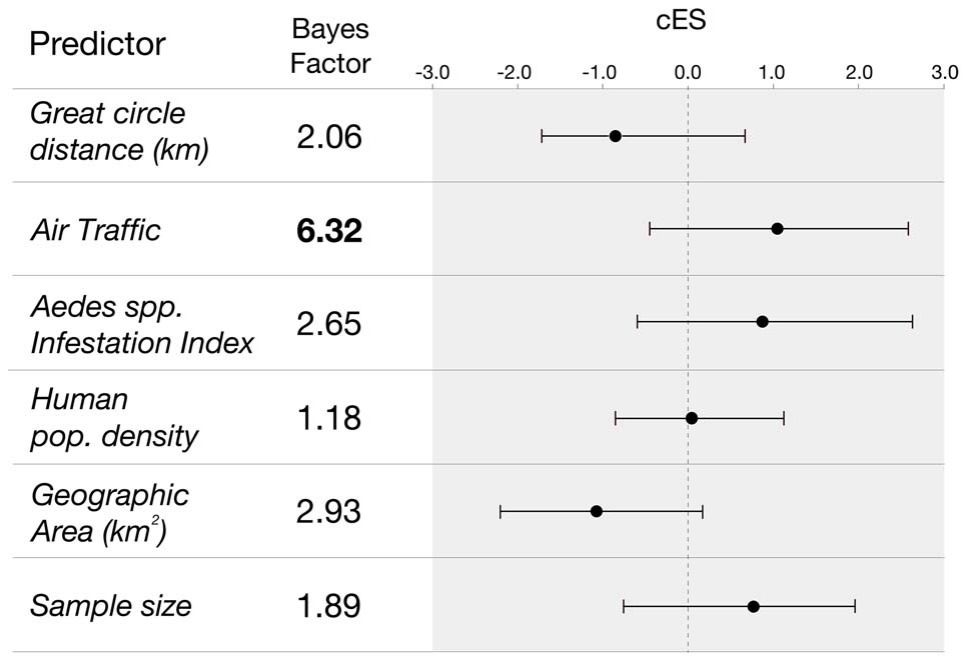
Predictors of DENV spatial dispersal. For each potential predictor, respective Bayes factor support and conditional effect sizes (cES) are shown. Circles and bars indicate respectively the mean and 95% Bayesian credible intervals of the estimated cES, respectively. Only predictors that obtained a Bayes factor support above 3 are considered significant (highlighted in bold).

One hundred and seventy-eight Brazilian genome sequences were used to determine the factors underlying dengue virus dispersal on a countrywide scale using the GLM spatial diffusion model. As candidate predictors of viral spread, we included geographic distances, national air traffic data, *Aedes aegypti* infestation densities, human population densities and spatial area of each considered location at a regional level (k = 5). To exclude the effect of potential sampling biases, we considered sampling sizes in our model. Figure 6 summarizes the Bayes factor support for each predictor and the corresponding conditional effect sizes on a log scale. Our results indicate a significant role of air traffic fluxes in viral spread between spatial regions (BF = 6.32, with a positive mean conditional effect size of 1.09 and Bayesian credible interval: −0.47, 2.45). Sample sizes did not attain a significant BF support, suggesting that sampling biases did not influence our conclusions.

## Discussion

We have characterized the introduction, establishment and drivers of dengue virus spread in Brazil using a combination of genetic and epidemiological data. By applying a flexible phylogeographic approach to full genome sequences, our analyses strongly support a human role for the spread of DENV through aerial transportation of humans and/or mosquito vectors. In particular, our results show that air traffic between geographical regions results in a modest but statistically significant Bayes factor support for the inclusion of this predictor in the model. Furthermore, the positive conditional effect size suggests that air traffic fluxes are positively associated with DENV dispersal. The results obtained using continuous diffusion models corroborate this hypothesis. Specifically, the average daily dispersal distances for DENV serotypes 1 to 3 are approximately 18 times larger than the mean dispersal distances measured for its main vector *Aedes aegypti* [44], [45].

Our GLM approach allows us to simultaneously estimate DENV phylogenetic diffusion processes and quantitate the contribution of potential predictors [41], [42]. Advantages include flexibility and the capacity to reduce the impact of sampling biases and fully exploit the geographic information embedded in the DENV genomic sequence data. Although population density has been shown to correlate with dengue virus dispersal over short distances such as are found in urban Viet Nam [46], we expected that this effect would be diluted in samples representing larger geographic areas such as those represented in our study, We also did not obtain significant support for the inclusion for sample sizes in the model, suggesting that our conclusions are not affected by sampling biases.

The hypothesis-testing framework used in this study was recently applied to investigate the impact of air traffic networks on influenza virus [15] and to capture the main drivers of cross-species transmission [41]. This framework focuses on quantitating associations between potential determinants of viral spread and virus migrations inferred from the phylogenetic history, rather than focusing on viral persistence in a particular location. Although *Aedes aegypti* mosquito infestation indices, an ecological proxy for vector incidence, does not appear to be significantly associated with DENV dispersal, we cannot rule out a role in the persistence or maintenance of the virus in particular areas. Structured coalescent models will be needed to rigorously evaluate candidate ecological and evolutionary aspects associated with dengue virus maintenance. Nonetheless, we speculate that environmental factors such as humidity and temperature, as well as the availability of susceptible natural invertebrate and vertebrate hosts for maintaining the viral cycle have most certainly facilitated the maintenance of dengue virus serotypes in the Americas [47]. Computational models that allow different evolutionary processes through time (epochs or seasons) will be critical for explicitly testing the impact of ecological factors with seasonal variation, such as humidity and temperature, in the dispersal of dengue at different spatial scales.

The epidemic profile of each dengue circulating serotype until 2008 indicated a strong concordance between rises in state counts and the estimated date of introduction of new lineages in Brazil. Our data also suggest that serotypes 1 and 2 are in-phase with each other and out-of-phase with the recently re-introduced serotype 4. However, the patterns observed for serotype 3 are more complex and additional genomic data and monthly incidence surveillance reports will be needed to elucidate its dynamics. In a study conducted in Bangkok, where the four dengue serotypes also co-circulate, within serotype lineage extinction and replacement processes were shown to occur in approximately 10-year intervals [48]. Our data shows a similar pattern in that lineage extinction and replacement occurred in Brazil at approximately 10 year-intervals for serotypes 1 and 3, and 7 year-intervals for serotype 2. Our approach takes advantage of the strong temporal structure present in dengue virus phylogenies [49] and focused on time-calibrated phylogenies from which accurate population dynamic models captured serotype-specific changes in effective population size over time. In line with our findings, mathematical modeling has previously predicted that the success rate of an invading lineage is lowest when disease prevalence peaks [50]. Future studies should investigate evolutionary markers that determine invasion dynamics of a successful lineage and, more specifically, the mechanisms underlying extinction and replacement of DENV serotypes and lineages in human and mosquito populations [48], [50]–[52]. We estimated evolutionary rates for serotype 2 lineages to be two to six times faster than those for serotype 1 and 3. Whether this reflects a fitness advantage of this strain as suggested in Southeast Asia [53] requires further investigation.

Our results using full genome sequence data confirmed that since 1985 multiple distinct lineages of DENV-1 [54] and DENV-3 [55] have been introduced in Brazil. We estimated that serotypes 1 to 3 have been introduced at least on eight distinct occasions, most probably from the Caribbean region (75%, 6/8) and Venezuela (25%, 2/8) (Figures 1 to 3). In a recent investigation of serotype 4 phylogeography in Brazil, we estimated 2 importations from the Caribbean and 3 importations from Colombia/Venezuela [15]. Unfortunately, data from serotype 4 lineages circulating in Brazil were insufficient for inclusion in the analyses described here. Taken together, our data suggest that future DENV lineages may be introduced from the Caribbean and/or countries bordering Brazil, into the northern areas Brazil before spreading countrywide. However, caution is needed when making predictions about viral emergence [56].. Indeed, the recent introduction of dengue serotype 4 genotype I from Southeast Asia into Brazil [15] is a reminder that new lineages from outside the Americas can be introduced in Brazil due to chance importation events.

Finally, because changes in deforestation may have a high impact in the incidence of vector-borne diseases [47], [57] and our results suggest that the northern area of Brazil (roughly equivalent to the Amazon region) has a higher likelihood of receiving and subsequently exporting the virus to other areas, it is important to evaluate the impact of deforestation in DENV incidence in Brazil.

In conclusion, our investigation of DENV serotypes 1 to 3 spatiotemporal dispersal indicates distinct introductions and co-circulation of distinct serotypes and genotypes, highlights the impact of air traffic fluxes in the spatial spread of DENV within Brazil and shows that the introduction of new lineages is followed by epizootic amplifications in 7 to 10 year cycles. We cannot discern the relative importance of air transport of infected humans or mosquitoes in this model. Nor can we be confident that our findings will extend to the dynamics of DENV circulation on a global scale. However, given the robustness of our data and the importance of DENV to public health, we believe a strong case can be made for focused research on the role of human mobility in DENV population dynamics and human disease.

## Supporting Information

Figure S1 Geographic location of DENV states from where the isolates were obtained. Positions in the map are represented by red balloons with black dot inside.
(PPTX)

Table S1 List of RACE primers used for recovering 5′ and 3′ UTR regions of DENV serotypes.
(XLSX)

Table S2 List of DENV genomes used for phylogenetic and phylogeographic analyzes according to the serotype (DENV-1, DENV-2 and DENV-3), accession number, year of isolation and geographic location.
(XLS)

Table S3 Number of DENV complete sequences used for phylogenetic and phylogeographic analyzes according to the geographic location, continent or region, and DENV serotype.
(XLSX)

Table S4 Model selection of the continuous phylogeographic model for DENV-1 dataset (a), DENV-2 dataset (b), and DENV-3 dataset (c).
(XLSX)
